# Adenovirus Reveals New Pathway for Cholesterol Egress from the Endolysosomal System

**DOI:** 10.3390/ijms21165808

**Published:** 2020-08-13

**Authors:** Cathleen Carlin, Danny Manor

**Affiliations:** 1 Department of Molecular Biology and Microbiology, Case Comprehensive Cancer Center, School of Medicine, Case Western Reserve University, Cleveland, OH 44106, USA; 2 Department of Nutrition, Department of Pharmacology, Case Comprehensive Cancer Center, School of Medicine, Case Western Reserve University, Cleveland, OH 44106, USA; dxm178@case.edu

**Keywords:** cholesterol, early 3 region, lipid transfer proteins, membrane contact sites

## Abstract

In addition to providing invaluable insights to the host response to viral infection, adenovirus continues to be an important model system for discovering basic aspects of cell biology. This is especially true for products of early region three (E3), which have provided the foundation for understanding many new mechanisms regulating intracellular trafficking of host cell proteins involved in the host immune response. Cholesterol homeostasis is vital for proper cellular physiology, and disturbances in cholesterol balance are increasingly recognized as important factors in human disease. Despite its central role in numerous aspects of cellular functions, the mechanisms responsible for delivery of dietary cholesterol to the endoplasmic reticulum, where the lipid metabolic and regulatory machinery reside, remain poorly understood. In this review, we describe a novel intracellular pathway for cholesterol trafficking that has been co-opted by an adenovirus E3 gene product. We describe what is known about the molecular regulation of this pathway, how it might benefit viral replication, and its potential involvement in normal cell physiology. Finally, we make a case that adenovirus has co-opted a cellular pathway that may be dysregulated in various human diseases.

## 1. Introduction

In addition to their vital function in the integrity of biological membranes, lipids play critical roles in multiple cellular functions including energy storage, cell signaling, and intracellular membrane trafficking [1,2]. Moreover, organelles within a cell have distinct lipid compositions, contributing to their unique identities and specialized functions [3]. For the most part, lipids are transported to various cellular membranes, where they may be subsequently modified, from their site of synthesis in the endoplasmic reticulum (ER) [4]. There are notable exceptions, for instance dietary cholesterol is delivered to multiple cellular membranes including the ER following receptor-mediated endocytosis of low density lipoprotein (LDL) particles [5]. Intracellular lipid trafficking is regulated by both vesicular and non-vesicular mechanisms [1,2], with non-vesicular transport being particularly important at tethered membrane contact sites (MCSs), comprising small cytoplasmic gaps (typically 10–20 nm) between ER membranes and virtually all other cellular organelles [6,7]. MCSs also have important roles in calcium and other signaling events, organelle inheritance, organelle division, and autophagy [8,9,10,11,12,13]. Recent studies have shown that many viruses co-opt MCSs to support their own replication within the infected host cell. For instance, multiple positive-strand RNA viruses, including poliovirus, hepatitis C virus, human rhinovirus, Aichi virus, and encephalomyocarditis virus, “hijack” MCSs to induce formation of viral replication complexes (VRCs) [14,15,16,17,18], and the late endosome/lysosome (LE/Lys) membrane protein NPC1 that mediates cholesterol transport to the ER serves as the intracellular receptor for Ebola and Marburg filoviruses [19]. Following a brief discussion of pertinent background information, we describe a novel host–virus interaction regulating cholesterol trafficking at the endosome–ER interface that provides new insights to physiological pathways regulating cholesterol homeostasis.

## 2. Non-Vesicular Lipid Transport at MCSs

**Figure 1:**
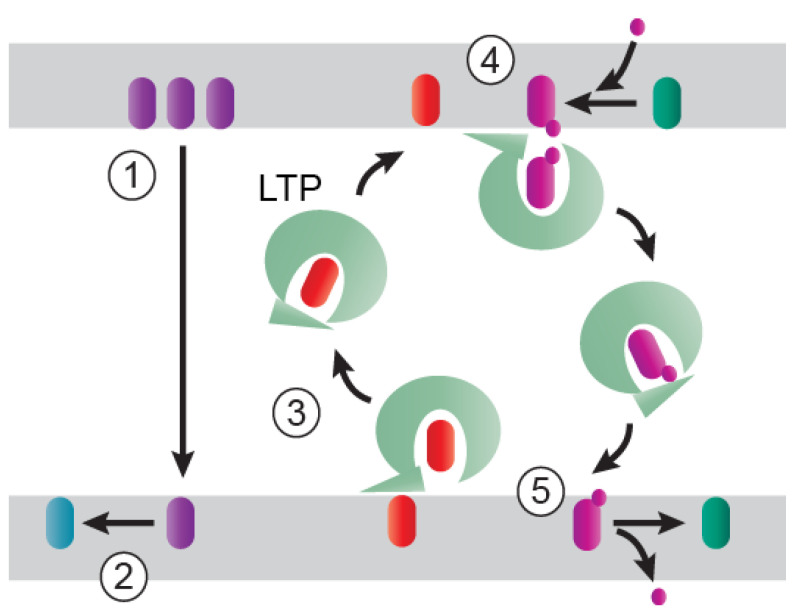
Potential mechanisms involved in non-vesicular lipid transport at membrane contact sites (MCSs). Spontaneous lipid transfer (1; purple lipid) could be aided by a sink capable of driving transport down a chemical concentration gradient by lowering the concentration of the transferred lipid in the acceptor membrane (2; blue lipid). Lipid transport could also be mediated by lipid transfer proteins (LTPs) that extract one lipid from a donor membrane (3; orange lipid) and deliver it in a sealed hydrophobic pocket to an acceptor membrane, where it is exchanged for a second lipid generated in the acceptor compartment (4; magenta lipid). In this model, LTP-dependent lipid transport is driven by a concentration gradient that is maintained by the continuous hydrolysis of the transported lipid in the donor compartment (5; green lipid).

Two mechanisms have been put forth to account for non-vesicular lipid transport at MCSs (Figure 1). First, lipid transfer could occur spontaneously, either by diffusion through the aqueous cytosol or transient hemifusion of apposing membranes [20]. However, in vitro spontaneous inter-vesicle transfer rates appear to be much too slow, up to several days, to be physiologically relevant [21,22]. There are notable exceptions, for instance spontaneous translocation of cholesterol occurs much faster, at the order of 1 to 3 h in vitro, as expected from its structure, which contains fewer hydrophobic carbons compared to other membrane lipids [22]. Additionally, metabolic activities associated with different ER enzymes that lower ER levels of free cholesterol, such as phosphatidylcholine biogenesis to absorb excess free cholesterol and formation of cholesteryl esters for storage in lipid droplets, could create cholesterol sinks capable of driving spontaneous cholesterol transport down a chemical concentration gradient until reaching equilibration [21,22]. A second mechanism involving high efficiency transport by lipid transfer proteins (LTPs) has gained favor over the past several years [23]. Although several families of LTPs have now been identified, members of the ORP (oxysterol-binding protein (OSBP)-related proteins) family are probably the best understood at a mechanistic level [23,24]. ORPs are characterized by the presence of an evolutionally conserved hydrophobic pocket, or OSBP-related domain (ORD), with dual capacity to bind PI(4)P and sterol or another lipid. Most ORPs possess additional conserved motifs, allowing them to form molecular tethers by simultaneously associating with two adjacent membranes at MCSs. Despite considerable structural variability of ORPs, a unifying mechanism for facilitating lipid transfer is beginning to emerge [25]. According to a current working model, LTPs pick up their lipid cargo from donor membranes when the lid covering its hydrophobic ORD is in an open conformation [26]. Sterols initially bind a sterol-binding domain (SBD) in the open lid domain before subsequently moving into the binding tunnel. The lid then assumes a closed conformation, allowing the lipid to be transported across MCSs in a sealed hydrophobic pocket until encountering an acceptor membrane. LTPs exchange the bound lipid for another lipid in the acceptor membrane, which is then transferred back to the donor membrane. This working model was derived from studies involving OSBP, which exchanges cholesterol synthesized in ER, for PI(4)P generated in the trans-Golgi network that was subsequently degraded by the ER-resident lipid hydrolase Sac1. According to this model, forward LTP-dependent lipid transport is driven by a concentration gradient that is maintained by the continuous hydrolysis of the transported lipid in the donor compartment, rendering the forward transfer irreversible and acting as a timer to halt transfer when hydrolysis becomes limiting. These studies revealed an unanticipated role for PI(4)P, previously known primarily for providing a molecular signature of organelle identity and function, in providing an energy source that drives lipid exchange. Models describing the lipid cycling properties of other related LTPs bear strong resemblance to those of OSBP, although there is variability in whether the protein picks up the lipid cargo directly from the membrane or from the cytoplasm after the cargo has dissociated spontaneously [27]. There is also evidence indicating that some LTPs act as lipid sensors with additional roles, for instance in cell signaling and gene transcription, or as lipid chaperones for presenting lipids to other proteins [28,29]. At the present time, it is unclear whether these alternative functions are mutually exclusive, or if LTPs can operate differently depending on the physiological state of a cell.

## 3. Cholesterol Homeostasis

**Figure 2:**
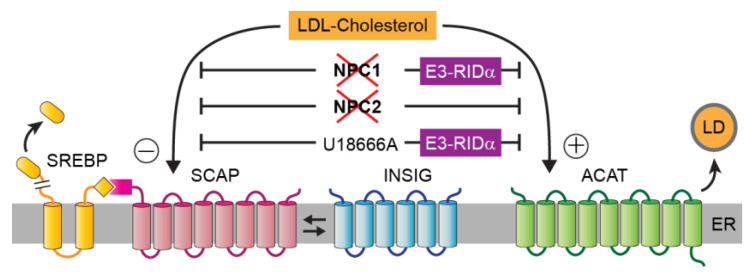
Endoplasmic reticulum (ER) cholesterol homeostatic machinery. Membrane-bound sterol regulatory element-binding protein (SREBP) precursors are retained in the ER through a tight association with the SREBP escort protein SCAP and the ER-resident protein INSIG. When ER cholesterol levels fall below a critical threshold level, SCAP dissociates from INSIG and mediates the COPII-dependent transport of SREBP precursor to the Golgi. Mature SREBP transcription factors are released by Golgi-resident proteases, and subsequently translocate to the nucleus where they activate genes involved in cholesterol uptake and de novo cholesterol synthesis. Excess-free cholesterol is stored in lipid droplets (LD) as acyl-CoA:cholesterol acyltransferase (ACAT)-generated cholesteryl esters. Negative SREBP feedback control and ACAT activity are both impaired in cells with reduced NPC1 and NPC2 function as well as following treatment with U18666A, a drug that phenocopies NPC by a direct interaction with the sterol-binding domain in NPC1. Ectopic RIDα expression is sufficient to restore ACAT activity, but not sterol regulation of SREBP, associated with reduced NPC1 function and U18666A treatment.

**Figure 3:**
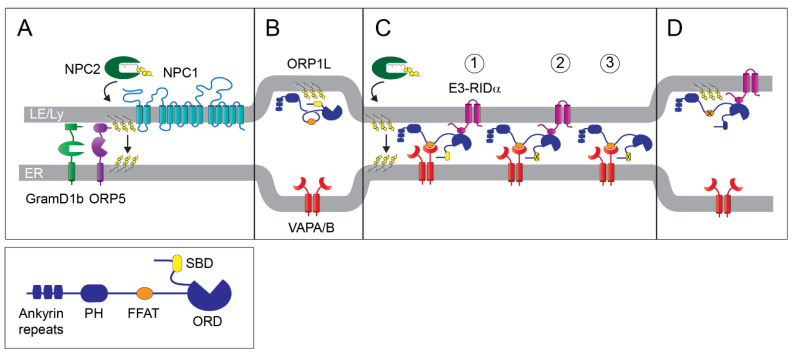
MCSs regulating cholesterol trafficking to the ER. (**A**) NPC1 appears to improve the kinetics of NPC2-mediated cholesterol insertion into late endosome/lysosome (LE/Lys)-limiting membranes, and to also participate in MCS formation through its interactions with ER membrane-anchored LTPs (GramD1b and oxysterol-binding protein (OSBP)-related protein 5 (ORP5)) that have both been shown to be essential for cholesterol transport. (**B**) ORP1L adopts a conformation that occludes its VAPA binding site in response to increasing levels of endosomal cholesterol. (**C**) Mechanisms that bypass NPC1 deficiency: (1) RIDα protein localized to endosomal membranes induces formation of ORP1L–VAPA MCSs that appear to mediate spontaneous cholesterol transfer to the ER following its insertion into limiting membranes by NPC2 under cholesterol-loading conditions. (2) The sterol-binding domain (SBD) in the ORP1L–ORD lid is dispensable in the RIDα pathway. (3) SBD-deleted ORP1L also reconstitutes ACAT-driven lipid droplet formation in NPC1-deficent cells independently of RIDα. (**D**) Mutations ablating the ORP1L FFAT (two phenylalanines (FF) in an acidic tract) binding motif inhibit ACAT-driven lipid droplet formation in NPC1-deficent cells with ectopic RIDα expression. **Inset:** ORP1L domain structure. PH, Pleckstrin homology; FFAT, two phenylalanines (FF) in an acidic tract; SBD, sterol-binding domain located in lid at entrance to oxysterol-binding protein (OSBP)-related domain (ORD).

Although cholesterol is synthesized in the ER, this organelle’s membrane is relatively cholesterol-poor, accounting for only 3–6% of total cellular cholesterol [30]. The molecular machinery that regulates cholesterol homeostasis via sterol regulatory element-binding protein (SREBP) transcription factors also resides in the ER (Figure 2) [31]. Cholesterol homeostasis is largely regulated by free cholesterol released during hydrolysis of endocytosed LDL particles in LE/Lys [32]. The cholesterol storage capacity of LE/Lys is regulated by the relative abundance of the unconventional phospholipid lysobisphosphatidic acid (LBPA) under the control of Alix, an LBPA-interacting protein involved in protein sorting into multivesicular body (MVB) LEs [33]. Subsequent cholesterol delivery to ER, either directly or via an intermediate stop at the plasma membrane, is regulated by two LE/Lys proteins: the multi-spanning limiting membrane protein NPC1, and the soluble protein NPC2 located within the vesicle’s lumen [34,35]. The current working model proposes that NPC2 mobilizes free cholesterol from internal LE/Lys membranes by a mechanism that has recently been shown to be critically dependent upon a direct interaction between NPC2 and LBPA [36,37]. Cholesterol is then transferred to a sterol-binding domain in the NPC1 amino terminus, via a direct interaction between NPC2 and a binding site in NPC1 that also regulates Ebola infectivity [38,39,40]. However, some details regarding the precise roles of NPC1 and NPC2 are still debated. For instance, in vitro studies indicate that NPC2 may be sufficient for transferring cholesterol into limiting membranes [41]. Similarly, NPC2 facilitates NPC1-independent transport of cholesterol from LE/Lys to mitochondria in collaboration with a member of the steroidogenic acute regulatory protein-related lipid transfer (START) protein family member MLN64 [42]. Suggested roles for NPC1 include chaperoning of cholesterol across the glycocalyx layer lining the internal LE/Lys perimeter, and accelerating NPC2-facilitated cholesterol insertion into limiting membranes [41,43]. NPC1 also appears to contribute to the creation of MCSs through its interactions with two LTPs anchored in ER membranes that different research groups have identified as essential for cholesterol transport (Figure 3A): the START family member GramD1b, and the oxysterol-binding protein family member ORP5, which has also been implicated in lipid exchange between the ER and plasma membrane [44,45,46]. Although these LTPs certainly meet the criteria of being strong candidates, how LDL cholesterol is exported from LE/Lys and delivered to the ER remains poorly understood.

NPC1 and NPC2 were originally identified since their mutated forms cause Niemann–Pick type C (NPC) disease, a severe and invariably fatal childhood neuropathy [47]. Cells with loss-of-function NPC1 and NPC2 mutations are characterized by the presence of enlarged perinuclear-clustered lysosomal storage organelles (LSOs) filled with unesterified cholesterol and other lipids. Since cholesterol is physically sequestered in these organelles, the ER sterol sensing machinery perceives the situation as deprivation, leading to compensatory activation of the SREBP-regulated cholesterol biosynthesis, thereby further elevating intracellular sterol levels and exacerbation of the pathology. In addition to de-repressed SREBP transcriptional activity, NPC1/2 mutations are associated with markedly reduced rates of cholesterol esterification, which is catalyzed by allosterically regulated ER resident acyl-CoA:cholesterol acyltransferase (ACAT) enzyme [48]. Additionally, NPC1 has been shown to be necessary for budding of lipid droplets, where cholesteryl esters are subsequently stored, providing an “escape valve” for cholesterol storage as well as a major nutrient reservoir, from the ER surface [49,50]. Cells also redirect cholesterol from plasma membrane to the ER for storage in lipid droplets in order to maintain homeostasis when cholesterol is abundant [51], a process that has been co-opted by enteroviruses to traffic cholesterol from the plasma membrane and extracellular medium to VRCs in support viral replication [52]. Although a majority of studies utilize ACAT-generated cholesteryl esters as a surrogate for LE/Lys-to-ER transport, there is evidence to suggest ACAT and SREBP are fed by different cholesterol pools and/or transport pathways in the ER [53]. LDL cholesterol accumulation in LE/Lys has also been shown to occur in NPC1-competent cells experiencing reduced cholesterol flux from organelles that are functionally integrated with LE/Lys, suggesting that NPC1 may facilitate a rate-limiting step for cholesterol transport to ER [54,55].

## 4. Mechanisms of LE/Lys Movement and Positioning

**Figure 4:**
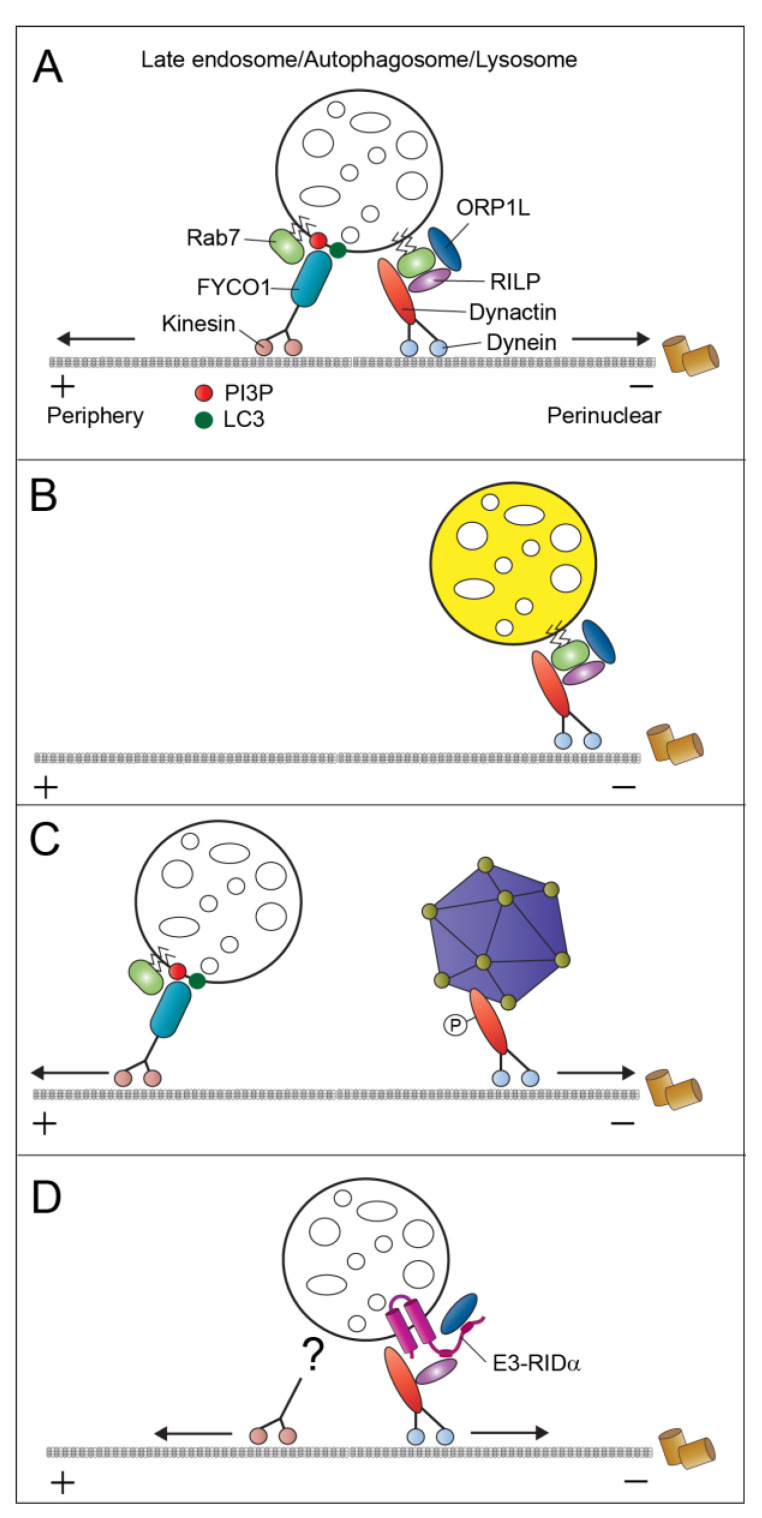
Mechanisms regulating dynamic LE/Lys motility. (**A**) Rab7 regulates bidirectional motility via engagement with kinesin and dynein motors through its effector proteins FYCO and RILP/ORP1L. (**B**) Excessive cholesterol causes LEs to become immobilized through stable recruitment of dynein motors. (**C**) Adenovirus-activated protein kinase A (PKA) phosphorylates light intermediate chain 1 (LIC1) causing dynein motors to shift from LEs to viral capsids. (**D**) RIDα reconstitutes RILP-dependent recruitment of dynein motors and uncouples LE from cholesterol regulation by alleviating the sterol-sensing activity of ORP1L. Mechanism mediating kinesin recruitment to RIDα-positive vesicles is currently unknown. “+”, plus end-directed; “−”, minus end-directed.

NPC1/2-mediated cholesterol trafficking has an additional essential role in regulating dynamic LE/Lys properties, allowing for continuous exchange of cargo in multiple intracellular transport pathways, including endocytosis, trafficking of Golgi-derived vesicles, and autophagy [56,57,58]. It is well established that LEs exhibit bidirectional motility on microtubules via engagement of molecular motors with opposing activities (Figure 4A) [59]. Minus end-directed movement towards the mitotic organizing center (MTOC), which promotes LE fusion with a relatively immobile pool of perinuclear lysosomes, is directed by cytoplasmic dynein–dynactin motors, whereas movement towards the cell periphery is mediated by plus end-directed kinesins. The interactions with molecular motors are regulated by the small GTPase Rab7 through its effector proteins RILP and FYCO respectively [60]. Importantly for this discussion, the Rab7–RILP–dynein–dynactin complex also associates with ORP1L, a member of the ORP family that senses cholesterol levels in LE/Lys-limiting membranes [61,62]. At low cholesterol levels, ORP1L has been shown to adopt a conformation, exposing an internal FFAT (two phenylalanines (FF) in an acidic tract) motif that interacts with the membrane-anchored ER protein VAPA at MCSs between LEs and the ER. This interaction disengages dynein–dynactin motors from Rab7–RILP complexes, leading to LE transport towards the cell periphery. At high cholesterol levels typically associated with inactivating NPC1/NPC2 mutations, the ORP1L FFAT motif is masked and LEs lose their dynamic properties because of stable recruitment of dynein–dynactin motors to Rab7–RILP complexes. Studies have also shown that cholesterol accumulation inhibits Rab7 membrane extraction by guanine nucleotide dissociation inhibitor, preventing it from engaging in multiple cycles of Rab7 activity governing efficient membrane trafficking in the LE/Lys pathway (Figure 4B) [63]. In addition to explaining the role of cholesterol in LE/Lys motility, these findings account for the abnormal LE/Lys-MTOC clustering phenotype and widespread trafficking defects typically seen in NPC1/NPC2-deficient cells.

## 5. Host Intracellular Trafficking Pathways Regulate the Early Stages of Adenovirus Infection

Group C adenovirus type 2 (Ad2) and Ad5 are lung pathogens whose primary targets are epithelial cells lining the upper respiratory tract [64]. Their efficient uptake involves sequential binding to coxsackievirus B adenovirus receptor (CAR) and αv integrin co-receptors [65]. Ad2 and Ad5 undergo stepwise uncoating programs initiated at the plasma membrane, which expose the viral lytic protein-VI that subsequently induces formation of small plasma membrane lesions [66,67]. These lesions allow for rapid and localized calcium influx and subsequent activation of a canonical repair mechanism involving lysosomal fusion with plasma membrane and release of acid sphingomyelinase (ASMase) into the extracellular space. Release of ASMase causes localized degradation of sphingomyelin in the outer plasma membrane leaflet, generating ceramide lipids that are known to cause inward invagination and endocytosis of damaged plasma membrane [68]. This host membrane repair process facilitates adenovirus internalization and escape of viral capsids from leaky endosomes to the cytosol [66]. Collectively, these early events induce the expression of various cytokines and inflammation-associated genes in innate cells such as macrophages and non-innate epithelial cell targets [69,70]. Viral capsids then undergo retrograde translocation to nuclear pores via a direct interaction with multi-subunit, minus-end directed cytoplasmic dynein motors (Figure 4C) in productively infected cells [71]. This process is mediated by virus-induced activation of protein kinase A (PKA), which phosphorylates the dynein light intermediate chain 1 (LIC1), causing the release of dynein motors from Rab7–RILP complexes and a partial cytoplasmic dispersal of LEs [72]. The PKA-mediated phosphorylation of LIC1 is also required for the binding of dynein to viral capsids and the expression of viral genes. In addition to identifying a novel form of host–virus competition, studies revealing the connection between PKA and dynein switching suggested adenovirus may have co-opted an as yet unidentified host mechanism for fine-tuning LE transport in response to PKA-regulated developmental and physiological cues. We have reported that viral infection is enhanced in respiratory epithelial cells with elevated levels of autophagy, which is an important adaptive response to high oxygen pressure in the airway [73,74]. Interestingly, recent studies have shown that the autophagy-related protein ATG16L1 and its binding partners ATG5 and ATG12 are required for plasma membrane repair, suggesting their possible involvement in adenovirus internalization and escape from leaky endosomes [75].

Studies carried out in cholesterol-depleted cells revealed that cholesterol also regulates adenovirus infection by multiple mechanisms [76]. Replenishment with exogenous cholesterol completely restored adenovirus internalization, but did not rescue viral gene expression, suggestive of roles for lipid-controlled trafficking or signaling. Interestingly, it has recently been shown that a population of lipid droplets buds directly from the inner nuclear membrane, which is contiguous with the ER, and that nuclear lipid droplets may function as platforms for regulating gene expression by scaffolding transcription factors [77]. These findings suggest a potentially unrecognized role for cholesterol trafficking and lipid droplet biogenesis in the control of viral gene transcription. Mechanisms derived from studying adenovirus may also shed light on factors contributing to coronavirus infectivity, where cholesterol has been shown to be important either through lipid rafts facilitating viral entry or by mediating viral fusion [78,79,80]. This cholesterol dependence provides a possible explanation for severe COVID-19 disease seen in individuals with underlying co-morbidities such as cardiovascular disease and diabetes as well as in older patients with elevated cholesterol levels, and suggest cholesterol lowering drugs as a potential therapy for critically ill patients [81,82].

## 6. Adenovirus E3 Protein Hijacks ORP1L to Restore Cholesterol Homeostasis

Among early viral gene transcripts, products of the early region three (E3) transcription region have proven to be especially interesting because of their ability to activate various cellular pathways that protect the host from viral-induced innate immune cytokine responses [70]. While initially beneficial, uncontrolled antiviral cytokine responses can ultimately cause tissue damage and potentially fatal adenovirus disease. The E3 protein RIDα is a small 13.7-kilodalton endosomal membrane protein originally identified because of its ability to alter EGF receptor (EGFR) trafficking. Specifically, we have shown that RIDα reroutes EGFR to degradative lysosomes in the absence of growth factor stimulation or receptor tyrosine kinase activity [83]. Our recent studies indicate that adenoviral expression of RIDα attenuates pro-inflammatory NFκB signaling associated with induction of stress-activated EGFR signaling during viral cell entry [84]. We have also shown that RIDα behaves as a Rab7 mimic, on the basis of its ability to reconstitute degradative receptor trafficking in Rab7-depleted cells (Figure 4D) [84,85]. The reconstituted pathway is regulated by interaction between RIDα and RILP by a mechanism dependent on the dynein-binding domain of this Rab7 effector protein [85]. Rather than accumulating at the MTOC, LEs are dispersed in cells infected with wild-type Ad2, suggesting the existence of redundant mechanisms for recruiting kinesin to LE/Lys [85,86].

We have also demonstrated an additional role for RIDα in cholesterol homeostasis. The observation that cells infected with a RIDα-null adenovirus mutant displayed an NPC-like phenotype characterized by perinuclear accumulation of aberrant lipid-filled LSOs provided the first hint of this activity [86]. Subsequently, we found that the infection-induced LSO phenotype was mitigated by an interaction between RIDα and the Rab7 effector protein ORP1L that supports LDL cholesterol transport to the ER (Figure 3C) [50]. In these studies, we were able to resolve spatial and temporal cholesterol flux by following the incorporation of oleic acid into cholesteryl esters in cells that had been pulse-labeled with radiolabeled LDL cholesterol. In contrast to its interaction with Rab7 involving amino terminal ankyrin repeats, the interaction between ORP1L and RIDα is regulated by sequences mapping to the exterior surface of the ORP1L–ORD that are not conserved in other ORP family members (Figure 3, inset) [49]. Our studies have shown that regulation of cholesterol trafficking by RIDα requires the previously described interaction between the ORP1L FFAT motif and the ER resident VAPA protein, which, as we have shown, occurs in uninfected cells with constitutive RIDα expression [49,50]. The RIDα/ORP1L pathway facilitates transport of LDL cholesterol by a mechanism that is sensitive to pharmacological ACAT inhibition, and is also associated with enhanced production of lipid droplets [49,50]. Strikingly, ectopic RIDα expression was sufficient to reverse cholesterol storage phenotypes in NPC1-deficient cells, although the RIDα-induced pathway does require NPC2 [50,86]. Similar to what is observed in infected cells, the RIDα-ORP1L pathway induces ACAT-dependent formation of lipid droplets in NPC1-deficient cells with constitutive RIDα expression [50]. However, this pathway does not restore negative feedback control of SREBP gene transcription, consistent with other findings showing that SREBP and ACAT may be subject to independent regulation [50]. In contrast to its FFAT motif, the SBD in the lid located at the entrance to the ORP1L hydrophobic pocket is dispensable for RIDα-induced cholesterol transport [50]. In fact, we found that an ORP1L construct missing the STD was fully functional in facilitating cholesterol transport to ACAT substrate pools in NPC1-deficient cells (Figure 3C) [50]. These findings have several important implications. First, ORP1L cholesterol sensing activity is critically dependent on its SBD in the hydrophobic pocket lid. Second, the interaction between RIDα and ORP1L likely counteracts negative effects of the ORP1L–SBD on cholesterol transport by reducing its accessibility to cholesterol in LE/Lys-imiting membranes. Third, ORP1L does not appear to be directly involved in cholesterol transport since the SBD is required to form a sealed hydrophobic pocket allowing the lipid to be transported across MCSs [87]. Our data support a novel working model, suggesting that protein contacts between ORP1L and VAPA facilitate spontaneous cholesterol transfer. We also postulate that this transport process is driven by a concentration gradient maintained by ACAT-driven formation of lipid droplets that lower concentrations of free cholesterol in the ER by acting as cholesterol ester sinks. Our data also suggest that the RIDα-induced pathway alleviates the cholesterol trafficking blockade in NPC1-deficient cells by enhancing the kinetics of NPC2-mediated cholesterol transfer to the LE/Lys-limiting membrane. However, we cannot exclude the possibility that cholesterol is actively transported by an as yet unidentified LTP located at ORP1L–VAPA MCSs, although a role of ORP5 has been ruled out [50].

In addition to further validation of this model, several open questions have not yet been resolved. First, how is the cholesterol storage defect incurred during viral cell entry? In this regard, it is well established that sphingomyelin and cholesterol interact strongly with each other at the plasma membrane, and that sphingomyelin depletion by ASMase causes a dramatic redistribution of cholesterol to the ER [88]. Thus, the membrane repair pathway induced by adenoviral protein VI plasma membrane wounding could conceivably create a bottleneck in NPC1-regulated movement of intracellular cholesterol, resulting in a cholesterol storage phenotype. The second outstanding question regards how this pathway benefits viral replication. At a minimum, restoration of cholesterol trafficking likely facilitates efficient intracellular trafficking and signaling of a myriad of cell surface receptors that regulate host immune function by preventing LE/Lys dysfunction in infected cells. In addition to stress-activated EGFRs, RIDα has been shown to be involved in down-regulating death receptors for tumor necrosis factor-related apoptosis-inducing ligands, allowing cells to evade proapoptotic responses to viral infection [89,90]. We have also found that ORP1L limits sensitivity to bacterially derived lipopolysaccharide signaling through toll-like 4 receptors, which are known to accumulate and become hyperactivated in LEs upon impairment of cholesterol flow [49,91]. However, this pathway may have additional functions that are yet to be discovered. For instance, lipids stored in lipid droplets could be mobilized for phospholipid synthesis during high demand for massive production of autophagic membranes, facilitating lytic release of new virions [92]. While our studies have focused on its cholesterol transfer activity, it is also possible that RIDα co-opts the ORP1L PI(4)P binding site, although this interaction remains controversial (see below). For instance, several studies have suggested that ORPs may act as PI(4)P-dependent scaffolds for assembling various signaling molecules such as G-protein-coupled receptors independently of their lipid transfer activities [93]. The fact that PI(4)P has functions that are independent of its role in lipid exchange is also illustrated by the role that this lipid plays in stabilizing viral RNA polymerase complexes responsible for synthesis of viral RNA at VRCs [94].

## 7. Are There Physiological ORP1L-Dependent Cholesterol Transport Pathways?

Several studies published since our original identification of this novel pathway suggest that adenovirus has captured a physiological pathway. The first example involves data demonstrating a requirement for ORP1L in maintaining proper cholesterol levels required for normal formation of MVB endosomes regulating sorting of membrane protein cargo to lysosomes for degradation [95]. It has also been reported that ORP1L-mediated cholesterol transport from its site of synthesis in the ER to a sub-population of EGF-stimulated MVBs contributes to down-regulation of EGFR signaling at MCSs tethered by annexin A1 and its calcium-dependent ligand S100A11 [96]. Both of these studies were carried out in ORP1L siRNA-depleted HeLa cells, and studies in EGF-stimulated cells also demonstrated a vital role for ORP1L–VAPA protein contacts. At first glance, this mechanism seems to be at odds with the LE/Lys-to-ER direction of cholesterol transport identified in our studies. However, cholesterol becomes sequestered in MVB intraluminal vesicles that essentially act as cholesterol sinks, owing to their rapid turnover in lysosomes [97]. Two other studies have found roles for ORP1L that mirror our findings in non-infected cells. One study reported that cholesterol esterification levels were reduced to the same levels in CRISPR-mediated ORP1L knockout cells, as seen in NPC1 knockout cells [98]. These investigators also provided evidence suggesting that cholesterol transport at ORP1L–VAPA MCSs required the dual sterol/PI(4)P-binding activity of the ORP1L–ORD. However, we believe that an OSPB-like lipid exchange mechanism is doubtful in this case for two reasons. First, since PI(4)P is synthesized primarily in the Golgi and plasma membrane [93], PI(4)P back-transfer to LE/Lys in exchange for cholesterol delivered to the ER seems unlikely. Second, recently published structural data suggest that ORP1L may not bind PI(4)P, although its membrane recruitment and cholesterol binding properties do appear to be regulated by interactions between PI(3,4)P_2_ and PI(4,5)P_2_ and basic patches on the ORD surface [99]. Consistent with our findings, another group has recently reported that that an ORD-deleted ORP1L construct with an intact FFAT motif was sufficient to restore cholesterol homeostasis in NPC1-deficient cells [45]. Altogether, these studies seem to indicate that ORP1L’s primary role may the creation of interorganellar membrane tethers via its interaction with VAPA, with the directionality of transport dictated by that of the cholesterol gradient. Adenovirus appears to have specifically captured ORP1L-VAPA contacts to facilitate transport of LDL cholesterol to ER, without impacting cholesterol levels required for normal MVB formation and MVB-mediated membrane protein degradation.

Adenovirus also has a long history of revealing novel insights to disease-associated pathways [69,100]. In our hands, ORP1L gene silencing did not have a significant impact on cholesterol esterification levels in NPC1/NPC2-competent cells, raising the possibility that the adenoviral RIDα protein has co-opted a pathway that is normally tightly regulated rather than constitutively active [49,50]. There are several human disease processes in which dysregulated activity of such a pathway might come into play. The first involves the transformation of macrophages to foam cells filled with lipid inclusions associated with chronic inflammation in a variety of metabolic, infectious, and autoimmune diseases [101]. Foam cells have been shown to have a multitude of roles contributing to pathological immune responses, making them interesting targets for therapeutic development [102]. Although details are still emerging, foam cell biogenesis has been studied extensively in atherosclerosis, where an imbalance of cholesterol influx, esterification, and efflux gives rise to cholesterol-laden foam cells with a critical role in the etiology of the disease [103]. Studies have demonstrated that the ER undergoes fragmentation, producing vesicles, which are highly enriched in ACAT1 activity and associate tightly with LEs, during foam cell formation associated with atherosclerosis [104,105]. Strikingly, macrophages with these structures effectively esterify LDL-derived free cholesterol in cells producing an NPC-like cholesterol storage phenotype resulting from treatment with U18666A, a cationic steroid that specifically inhibits NPC1 [106]. In fact, induction of the ACAT1–LE pathway led to a significant recovery of cholesterol esterification in macrophages with an NPC1 mutation, and also extended the lifespan of NPC1-deficient mice, suggesting up-regulated expression of an alternative route for cholesterol transport. The authors of these studies speculate that that the induced ACAT/LE structures are specialized for cholesterol esterification, but the molecular basis of cholesterol transfer between these tightly apposed organelles is currently unknown. Our studies suggest a possible role for ORP1L, which is known to be highly expressed in macrophages and to also be up-regulated upon the differentiation of human monocytes into macrophages [107], in pathological accumulation of cholesteryl esters. Interestingly, foam cells are enriched in either triacylglycerol or cholesteryl esters, depending on the inflammatory or pathogen signals driving their differentiation, suggesting that ORP1L may represent a common therapeutic target for diseases capable of inducing formation of foam cells associated with excessive cholesteryl ester production [101]. A second possible scenario where an ORP1L-dependent cholesterol transport pathway might be mis-regulated, involves production of lipid droplets that aberrantly accumulate as a consequence of nutrient and oxidative stress in various cancers [108]. Several studies have suggested that this pathway is regulated by “hyperactive” Akt-dependent SREBP signaling, leading to up-regulated expression of the LDL receptor and subsequently enhanced LDL uptake [109]. Since NPC1 is known to be rate-limiting in the canonical cholesterol transport pathway, it would not be surprising to find elevated activity of an alternative mechanism capable of delivering LDL-derived free cholesterol to the ER that is NPC1-independent. Clearly, understanding the mechanisms by which adenovirus co-opts an ORP1L-mediated cholesterol transport pathway that might be misappropriated in various pathological settings warrants further investigation.

## 8. Conclusions and Future Directions

We have discovered a previously unrecognized pathway for maintaining cholesterol homeostasis that is independent of NPC1 and relies on the interaction between ORP1L on LE/Lys and VAPA in ER membranes. The mechanism is also novel in that cholesterol transport appears to occur spontaneously as a consequence of ACAT-driven formation of lipid droplets that act as sinks to absorb excess free cholesterol in the ER. This hypothetical mechanism is consistent with multiple lines of evidence indicating that ORP1L provides a flexible platform supporting bidirectional cholesterol transport at different types of MCSs. We postulate that adenovirus has captured a physiological pathway, allowing for a rapid and reversible expansion of cholesterol esterification capacity under conditions when NPC1 becomes rate-limiting. This “bypass” route serves to enable the virus to overcome deficits in cholesterol trafficking incurred during the early stages of the replicative life cycle. The mechanistic details that regulate cholesterol transport to the ER homeostatic regulatory pools remain poorly understood. Given the central role of cholesterol in viral replication, it will now be important to determine if this pathway is a common target for multiple viruses acting through different mechanisms, and if so, whether this information can be used to construct a more detailed understanding of cholesterol homeostasis in health and disease. The ability of the adenovirus RIDα protein to overcome inhibition of ORP1L–VAPA complex formation by high endosomal cholesterol content via a protein interaction with the ORP1L–ORD also provides important clues regarding how this pathway might be co-opted by pathological and infectious stimuli. Our studies suggest it may be desirable to develop therapeutic strategies capable of switching ORP1L cholesterol transport activity on or off, in order to correct disease-specific imbalances of cholesterol influx and esterification.

## Abbreviations

ACAT	Acyl-CoA: cholesterol acyltransferase
ASMase	Acid sphingomyelinase
CAR	Coxsackievirus B adenovirus receptor
E3	Early region three
EGFR	EGF receptor
ER	Endoplasmic reticulum
FFAT	Two phenylalanines (FF) in an acidic tract
LBPA	Lysobisphosphatidic acid
LDL	Low density lipoprotein
LE/Lys	Late endosome/lysosome
LIC1	Light intermediate chain 1
LSO	Lysosomal storage organelles
LTP	Lipid transfer protein
MCS	Membrane contact sites
MVB	Multivesicular body
MTOC	Mitotic organizing center
NPC	Niemann–Pick type C disease
ORD	OSBP-related domain
ORP	Oxysterol-binding protein (OSBP)-related protein
PH	Pleckstrin homology
PKA	Protein kinase A
SBD	Sterol binding domain
SREBP	Sterol regulatory element-binding protein
START	Steroidogenic acute regulatory protein-related lipid transfer
VRC	Viral replication complexes
